# Response: Commentary: Acetaminophen Enhances the Reflective Learning Process

**DOI:** 10.3389/fpsyg.2020.02099

**Published:** 2020-09-03

**Authors:** Jason Shumake, Rahel Pearson, Seth Koslov, Bethany Hamilton, Charles S. Carver, Christopher G. Beevers

**Affiliations:** ^1^Department of Psychology, University of Texas at Austin, Austin, TX, United States; ^2^Department of Psychology, University of Miami, Coral Gables, FL, United States

**Keywords:** acetaminophen, comment, COVIS, reflexive learning, reflective learning

We are writing in response to a commentary by McPhetres (2019), who misrepresented our work regarding the differential impact of acetaminophen on reflexive vs. reflective learning (Pearson et al., 2018). His commentary begins with a summary that fails to discuss or even acknowledge our actual theoretical framework. Instead, McPhetres discusses an unreasonable extrapolation of our findings that he has *imagined*: namely, a “smart pill” that improves learning writ large.

Our actual research hypothesis and findings are not remotely this extraordinary. On the contrary, they are grounded in theory emphasizing the contribution of serotonin to the balance between two modes of self-regulation, namely the Competition between Verbal and Implicit Systems model (COVIS) of learning and decision making. Our hypothesis was that serotonin—not acetaminophen *per se*—biases neural systems toward reflective learning at the expense of reflexive learning. Acetaminophen was a convenient tool for manipulating serotonin without the logistical hurdles of administering a prescription SSRI, with the obvious caveat of having non-serotonergic effects as well.

McPhetres goes on to use selective quotations to support his thesis that our research implications are “severely overstated.” One of these is “… acetaminophen could potentially help people make difficult decisions by reducing emotional responses to affective contexts while at the same time facilitating more deliberative, effortful information processing…” The full quote from our paper makes clear that this idea originates from another study and that readers should regard it as speculation:

“Although speculative, acetaminophen could potentially help people make difficult decisions by reducing emotional responses to affective contexts while at the same time facilitating more deliberative, effortful information processing (DeWall et al., 2010)” (p. 1,033).

Another is, “We found that reflective-optimal decision-making can be enhanced by acetaminophen.” McPhetres fails to give us credit for the sentences that immediately follow this ‘‘overstatement”:

“It is important to note that some of the analyses yielded non-significant results (e.g., overall accuracy for the reflective-optimal task did not differ between groups) and that other findings (e.g., acetaminophen reduces the likelihood that criterion was met for the reflexive-optimal task) emerged from exploratory analyses. Thus, these findings should be considered preliminary and need to be interpreted with caution until they are replicated” (p. 1,033).

We fail to see how one could interpret this (the bulk of the concluding paragraph) as overstating anything, much less as an encouragement to take Tylenol as a “smart pill.” Moreover, we found that acetaminophen does not improve and may worsen information integration (reflexive learning)—something McPhetres forgot to mention.

On top of misrepresenting our conclusions, McPhetres makes several factual errors (see also Table 1):

1. He accuses us of misreporting eta-squared values, confusing *generalized* eta-squared (what we calculated) with *partial* eta-squared (what he calculated). (Note the subscript “p” for eta-squared in his formula and the absence of said subscript in our publication.)
2. He mistakenly lists “depression scale” as an additional measured variable and “low score on depressive symptoms” under “vague exclusion criteria.” In fact, not being depressed (i.e., below a pre-determined cut-off score) was part of the *eligibility screen to enroll in the study*.
3. Out of 100 participants, 6 were excluded for protocol violations (not starting task 60–75 min after treatment administration or, in one case, sleeping during the task), and 7 were excluded because their mean performance was at or below chance responding, a customary exclusion criterion for eliminating random responders. Contrary to what McPhetres imagines, we did not drop these participants to lower our *p*-values. In the interest of transparency, we reran all analyses with all available data. None of our conclusions would have been altered by the exclusion criteria. In fact, most *p*-values would have been *smaller* had we included all participants. The statistical code and output for both original and updated analyses, along with an extensive explanation of every step in the data analysis, are documented in the following blog post: https://jashu.github.io/post/apap/.
4. McPhetres falsely asserts that trials-to-criterion and learning-rate analyses were exploratory (alleged examples of HARKing), so he apparently regards accuracy as our only confirmatory outcome metric. In fact, trials-to-criterion and learning curves—not accuracy—were the only outcomes used in our prior work (Maddox et al., 2015). Why would McPhetres consider two metrics with prior empirical support to be “exploratory” and the completely new metric to be “confirmatory?” Clearly, we always planned to test our hypothesis with all three approaches, regardless of the *p*-value obtained for any single one.

**Table 1:** Reproduction of table from McPhetres (2019) with our response to each point raised.

**Degree of freedom code (Wicherts et al., 2016)**	**McPhetres (2019) criticism**	**Our response**
T2: Vague hypotheses	• “We anticipated that acetaminophen would enhance effortful, reflective learning, and decrease reliance on intuitive, reflexive learning strategies” • “Enhanced” and “poorer” performance	As reviewed in our paper (Pearson et al., 2019), this “vague” hypothesis was derived from and constrained by prior theoretical neuroscience models and predicts that the probability of success will increase faster for the acetaminophen group on a reflective learning task, and it will increase slower for the acetaminophen group on a reflexive learning task.
D5: Measuring additional variables	• Depression scale • Task performance at “chance level” • Trials to criterion, learning rate	There was only one measured variable that was analyzed: correct response, which was assessed for 150 trials on each of two learning tasks. McPhetres is listing here study eligibility criteria, data quality criteria, and analytic approaches—not measured variables.
D6: Lack of power analysis	• No justification for sample size given	We used text from Mischkowski et al. (2016) (p. 1,346), which suggests 40–54 participants per cell provides sufficient power to detect a behavioral effect of acetaminophen. Ours was not a pure between-subjects design but rather a between-subjects factor acting on a within-subjects difference.
D7: No Sampling plan specified	• No sampling plan specified	Sampling plan, based on the above reference, was to recruit 50 participants per group.
A1/2: Vague exclusion criteria and “datacleaning”	• Low score on depressive symptoms • Lacking “complete” task data • Task performance “at or below chance”	Depression scores were part of study eligibility prescreen; no one who completed the study was excluded for this. Eliminating random responders is standard practice, not a researcher DF. Reanalysis including all participants does not weaken findings.
A3: Treating statistical abnormalities *ad-hoc*	• Because of possible suppression “… exploratory analysis was conducted to examine accuracy until the first rule change” • Recoding trials to criterion as Yes/No “since the majority of participants failed to reach criterion …”	Analysis of the first rule has a strong *a priori* theoretical rationale. This is explained in the following blog post: https://jashu.github.io/post/apap/. An analysis of variance requires variance to analyze. When the majority of participants are right censored (their actual trials-to-criterion value was not observed and is unknown), dichotomizing to event observed/not observed is the best analytic option.
A6: Multiple scorings of the DV	• Overall accuracy score • Accuracy until the first rule change • Number of trials to criterion • Dichotomizing reaching criterion (Yes/No) on the reflexive-optimal task	These are indeed multiple scorings of the DV, but they are important alternative perspectives to consider, and we obviously have reported all of them. Findings are largely consistent, regardless of how DV is operationalized.
R1: Reproducibility is not assured	• Data is not publicly available and not shared	Data are available here: Pearson et al. (2019)
R5: Misreporting results	• Eta-squared values are calculated incorrectly (noticed by a reviewer)	McPhetres and his reviewer calculated *partial* eta-squared, not eta-squared.
R6: Presenting exploratory results as confirmatory (HARKing)	• Trials to criterion scores • Learning rate analysis	These are not exploratory results; they are alternate constructs of the DV, but they all test the same *a priori* hypothesis. No new hypothesis was generated by any of these results.

We agree with McPhetres that these results need to be replicated; indeed, we say so in the article. But that would still be true if all our *p*-values were less than 0.005, or if none of them was less than 0.05. *P*-values from one study alone never provide strong evidence either for or against a hypothesis—period. But we disagree that we should have replicated all findings prior to publication. It would be different had we found something dramatic and unexpected (e.g., that acetaminophen increases GPA), or if we were advocating something ridiculous (e.g., that acetaminophen be used as a study aid). What we find baffling is that McPhetres seems to believe that if the reported effects are true then these incredible applications would follow, hence his concern that lay persons will overdose on Tylenol in an attempt to make themselves smarter. Even if all our findings replicate perfectly, they do not begin to support such outlandish applications.

We tried to test a theory about serotonin modulation of learning systems, and we tried to conduct reasonable analyses to inform that theory. Our only regret is that we did not make publicly available our data and statistical code at the time of publication, which left a vacuum for one reader's imagination to run wild. We believe in open science and reproducible research, and we encourage interested readers to visit our repository (Pearson et al., 2019) and evaluate our data for themselves.

## Author Contributions

JS wrote the first draft of this commentary and all authors provided critical revisions. All authors approved the final version of the commentary for publication. JS also wrote a blog post to complement this commentary, which, in addition to reproducing the published analyses, also details several analysis-related issues associated with the original study. This blog is available at: https://jashu.github.io/post/apap/.

## Conflict of Interest

The authors declare that the research was conducted in the absence of any commercial or financial relationships that could be construed as a potential conflict of interest.
